# Atypical Presentation of Burkitt Lymphoma With Isolated Peritoneal Involvement and Association With Refractory Type B Lactic Acidosis and Hypoglycemia Secondary to the Warburg Effect

**DOI:** 10.7759/cureus.35521

**Published:** 2023-02-27

**Authors:** Aymen K Abbas, Ahmed Y Osman

**Affiliations:** 1 Internal Medicine, Sheikh Khalifa Medical City, Abu Dhabi, ARE

**Keywords:** non-hodgkin's lymphoma, unexplained ascites, burkitt lymphoma, warburg effect, lactic acidosis

## Abstract

Lactic acidosis is considered to be one of the most common causes of high anion gap metabolic acidosis in hospitalized patients. Warburg effect can present with type B lactic acidosis and is considered to be a rare but well-known complication of hematological malignancies. Here, we present the case of a 39-year-old male who had type B lactic acidosis and recurrent hypoglycemia secondary to newly diagnosed Burkitt lymphoma. This case highlights the importance of considering malignancy workup in any case of unexplained type B lactic acidosis with vague clinical presentation, which can aid in early diagnosis and management.

## Introduction

Lactic acidosis is defined as a serum lactate concentration higher than 4 mmol/L and is considered to be one of the most common causes of high anion gap metabolic acidosis in hospitalized patients [1]. Etiology can be divided into reduced tissue oxygenation (type A) and conditions where hypoperfusion is not apparent (type B). Among many others, malignancy is a known rare cause of type B lactic acidosis [2]. Moreover, the Warburg effect is an uncommon but serious condition that happens when neoplastic cells shift to aerobic glycolysis and it is highly linked to many cancers, particularly B-cell lymphomas [3,4]. It is considered an oncological emergency and is associated with poor prognosis and a high mortality rate if untreated [4].

## Case presentation

A previously healthy 39 years old Asian male presented to the emergency department with abdominal distension for a three months duration. Two weeks earlier, he was seen in another hospital where he had a normal colonoscopy and underwent ascitic fluid drainage of 2.7 liters without reaching a diagnosis. His abdominal distension was getting worse gradually after discharge, and he started to develop exertional dyspnea as well. It was associated with night sweats, loss of appetite, and significant weight loss.

On admission, the patient was afebrile, blood pressure was 135/101 mmHg, pulse rate of 114 beats/minute, respiratory rate of 26 breaths/minute, and oxygen saturation of 98% on room air. Physical examination showed a soft, non-tender moderately distended abdomen with a positive shifting dullness sign. There was no organomegaly, chronic liver disease, or coagulopathy signs. His cardiopulmonary examination was normal. Laboratory studies showed normal electrolytes, creatinine, urea, lipase, liver function test, and coagulation panel. His random serum glucose was 3.5 mmol, serum albumin was 28 g/L, and serum total protein was 64 g/L. White blood cell count of 8.5 x 10^9^/L; neutrophils, 68.9%; lymphocytes, 23.5%; hemoglobin of 148 g/L; and platelet count of 391 x 10^9^/L. The C-reactive protein of 47.6 mg/L and negative COVID-19 PCR (coronavirus disease 2019 polymerase chain reaction) test. Venous blood gases showed Ph (potential hydrogen) of 7.36, HCO_3_ of 20 mmol/L, and lactic acid of 5.1 mmol/L. HIV (human immunodeficiency virus) and hepatitis panel negative.

**Figure 1:**
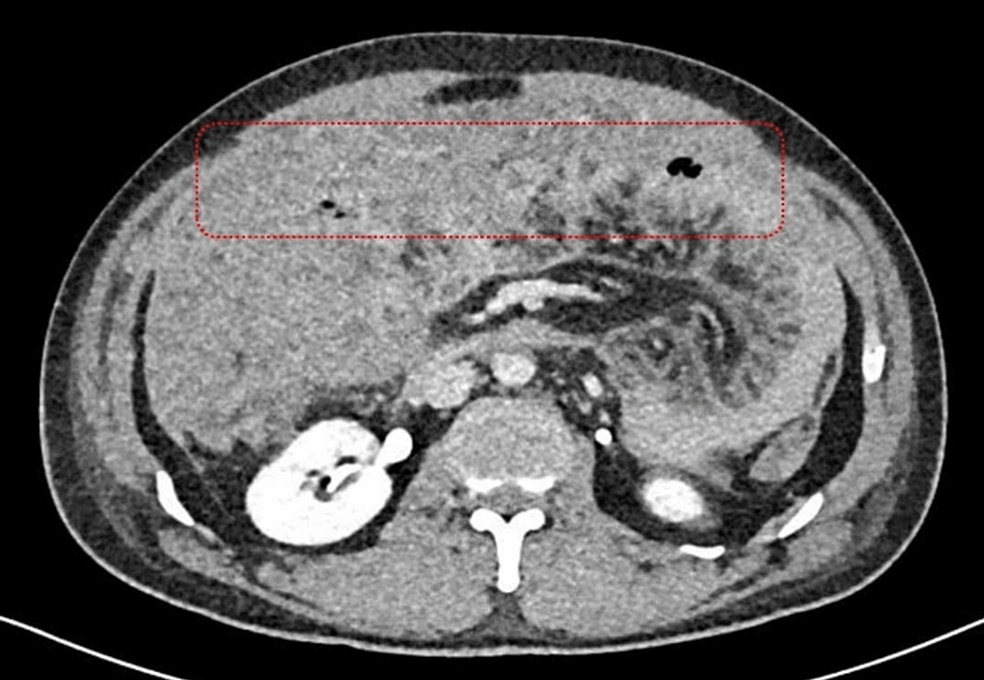
Computed tomography scan showing diffuse thickening of the omentum (omental caking)

**Figure 2:**
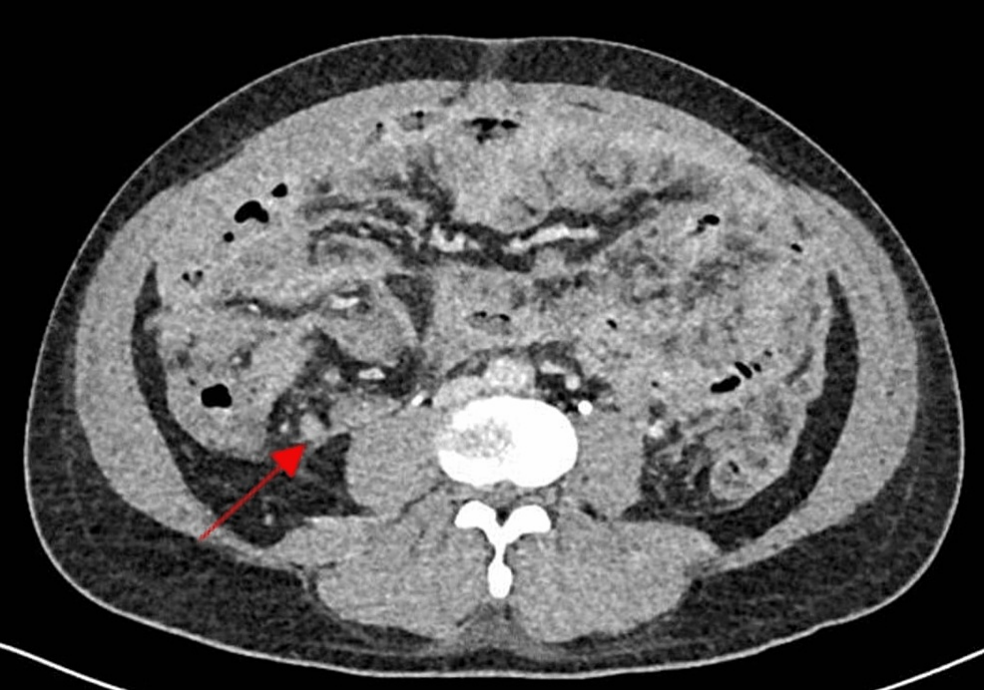
Computed tomography scan showing distal ileum bowel wall thickening

Computed tomography (CT) of the abdomen/pelvis with contrast ruled out bowel ischemia but showed significant diffuse thickening of the omentum (omental cake) and the peritoneum (Figure 1). Along with a marked 10 cm segment of the distal ileum bowel wall thickening, in the right lower quadrant, with an increase in enhancement and surrounding large volume of free fluid that is extending to all quadrants (Figure 2). The adjacent terminal ileum shows a reactive inflammatory change and diffuses peritoneal enhancement. Multiple mesenteric reactive lymph nodes with no lymphadenopathy.

Abdominal fluid analysis revealed white-pinkish purulent fluid with Ph (potential hydrogen) of 8, nucleated cells 104,239.00x10^6/L, neutrophils 5%, lymphocytes 15%, monocyte 10%, mesothelial cells 70%. Albumin was 22 g/L, protein 37 g/L, glucose 0.2 mmol/L, LDH (lactate dehydrogenase) 9,867 IU/L, triglyceride 0.65 mmol/L, ADA (adenosine deaminase) 121 U/l, and peritoneal fluid cytology showed few atypical cells that are of uncertain clinical significance.

Initially was treated empirically as a case of peritoneal tuberculosis (as per infectious disease team advice) along with intravenous hydration. Despite that, the patient continued to deteriorate with his lactic acid reaching 20 mmol/L, bicarbonate of 4 mmol/L, persistent hypoglycemia, and positive serum ketones. The patient was shifted to the intensive care unit where he had cardiac arrest secondary to hyperkalemia (K+ of 8 mmol/L) which was aborted after successful resuscitation.

**Figure 3:**
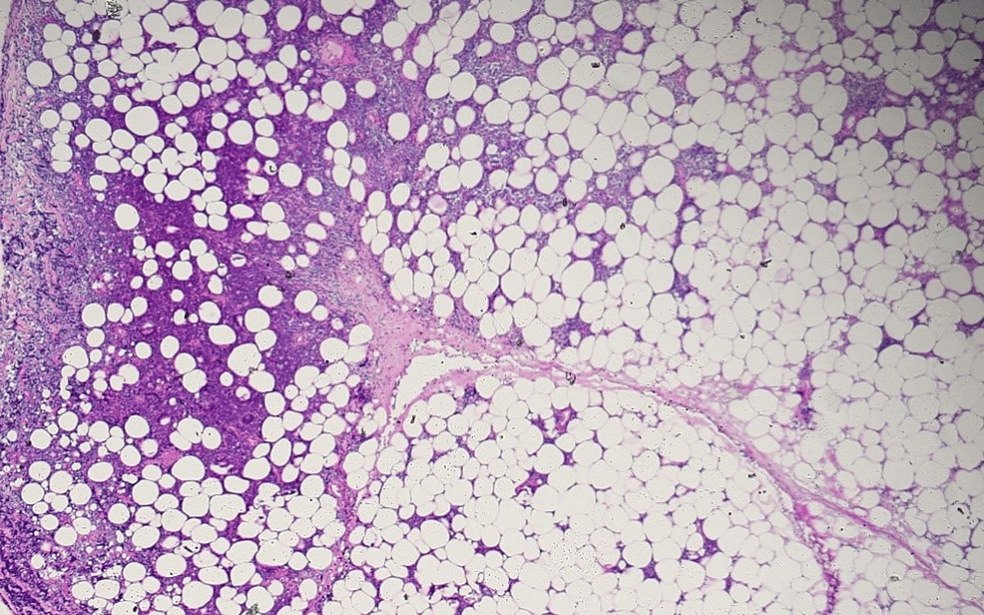
Omental biopsy with infiltration by lymphoma

**Figure 4:**
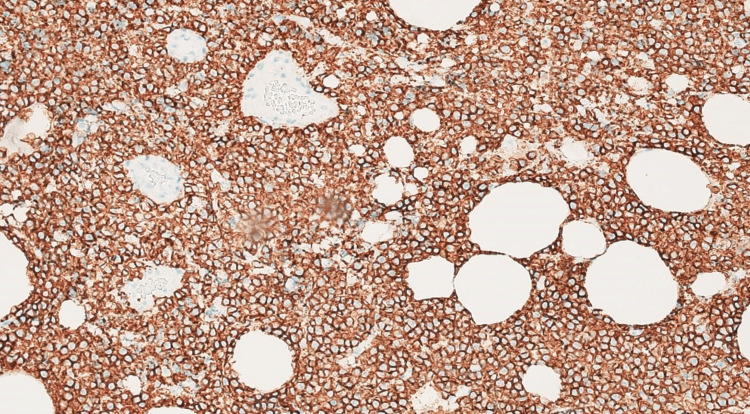
CD20 positive neoplastic cells

**Figure 5:**
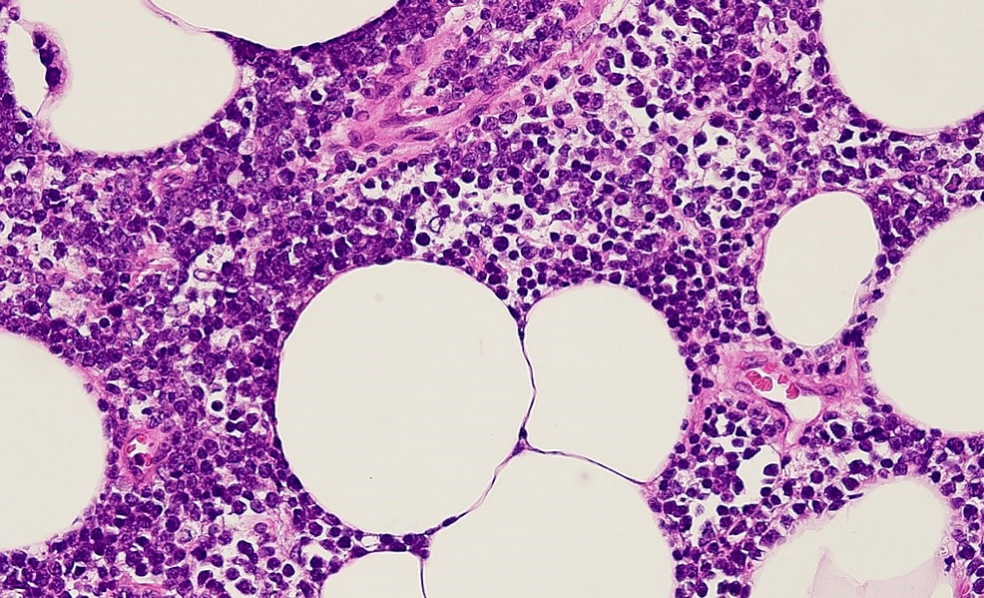
Lymphoma cells with admixed tangible body macrophages

A few days later, the surgical team was able to obtain an omentum biopsy sample which showed infiltration by diffuse sheets of monomorphic appearing intermediate-sized abnormal cells with the following immunophenotype CD20+/PAX5+ and CD10+; and are negative for CD3, CD5, BCL2, MUM1, CD30, CD34, and Cyclin D1 (Figures 3-5). The abnormal lymphoid cells are negative for TdT (Terminal deoxynucleotidyl transferase) immunostain. EBER ISH stain was negative for EBV (Epstein-Barr virus). C-MYC showed equivocal staining in the lesional cells. CD21 and CD23 immunostains do not show any discreet FDC meshwork. The Ki-67 proliferation fraction is very high, approximately >95% in the lesional cells.

FISH (fluorescence in situ hybridization) study showed t(8;14) MYC/IGH fusion in approximately 84% of nuclei. No rearrangement of BCL2 or BCL6 was observed (Table 1).

**Table 1:** FISH study showing t(8;14) MYC/IGH translocation FISH: fluorescence in situ hybridization

Abnormality Name	Result	Abn %	Cutoff %
8q24.1 (MYC sep)	Abnormal	91	<7.0
t(8;14) MYC/IGH fusion	Abnormal	84	<5.0
18q21 (BCL2 sep)	Normal		<9.0
3q27 (BCL6 sep)	Normal		<6.0

The findings were consistent with Burkitt lymphoma. The patient was then transferred to the hospital with oncology service and he passed away one day later as he had another cardiac arrest secondary to another hyperkalemic episode.

## Discussion

Lactic acidosis is divided into two subtypes according to pathophysiology type A which is more common and results from tissue hypoxia and low perfusion [5], and type B which happens in conditions with normal perfusion and is usually a result of toxins or drugs that alert cellular metabolism or due to nutritional deficiency that leads to mitochondrial dysfunction [6].

Malignancy although rare but considered to be a life-threatening cause of type B lactic acidosis [2,5]. Hematological malignancies account for the majority compared to solid organ malignancies [7]. The exact underlying mechanism is not well understood and is likely to be multifactorial [2]. Warburg effect also termed aerobic glycolysis was first described by the German physician Otto Warburg in 1923 [8].

Because of their higher proliferative rate compared to normal cells, tumor cells tend to increase their metabolism of glucose to lactate despite the presence of adequate oxygen. Under this condition, the conversion rate of glucose to lactate is around 100 times faster than through the citric acid cycle resulting in similar ATP (adenosine triphosphate) production over a similar period [9,10]. The association between lactic acidosis and hypoglycemia is uncommon but well-documented in some malignant causes, especially in lymphoproliferative tumors such as lymphomas [11].

In this case, the history of night sweats, weight loss, and generalized fatigue can be explained by B symptoms. The patient had refractory lactic acidosis that was not explained by hypoperfusion, toxins, or drugs. Omental biopsy and FISH study confirmed the diagnosis of Burkitt lymphoma. The rare combination of type B lactic acidosis, refractory hypoglycemia, ketoacidosis, and Burkitt lymphoma that presented as isolated peritoneal involvement was not reported in the literature previously.

The treatment approach for type B lactic acidosis should concentrate on treating the underlying cause, and the effect of acid correction and intravenous fluid rehydration treatment is not optimal [12].

The mortality rate from lactic acidosis secondary to malignancy is very high. Previously reported data showed that >70% of cases with lymphoma-associated Warburg effect died within 30 days [4,13,14].

## Conclusions

Warburg effect is a rare but very serious complication of hematological malignancies. Workup for lactic acidosis should rule out common etiologies and at the same time consider critical ones, especially in refractory cases. In any patient who presents with lactic acidosis and hypoglycemia with possible or confirmed lymphoma, the Warburg effect should be in the differential diagnosis. As in many other medical emergencies, time to treatment initiation is considered a significant factor in determining prognosis.
